# Supplementary material for: Exposing the molecular heterogeneity of glycosylated biotherapeutics
Source: Nat Commun. 2024 Apr 16;15:3259. doi: 10.1038/s41467-024-47693-8 (PMC11021452; doi:10.1038/s41467-024-47693-8)
Supplement: Supplementary file 2 — Reporting Summary [file 41467_2024_47693_MOESM2_ESM.pdf]

nature portfolio

Corresponding author(s): Wendy Sandoval

Last updated by author(s): Mar 28, 2024

Reporting Summary

Nature Portfolio wishes to improve the reproducibility of the work that we publish. This form provides structure for consistency and transparency in reporting. For further information on Nature Portfolio policies, see our Editorial Policies and the Editorial Policy Checklist.

Statistics

For all statistical analyses, confirm that the following items are present in the figure legend, table legend, main text, or Methods section.

n/a	Confirmed	
☐	☒	The exact sample size (*n*) for each experimental group/condition, given as a discrete number and unit of measurement
☐	☒	A statement on whether measurements were taken from distinct samples or whether the same sample was measured repeatedly
☒	☐	The statistical test(s) used AND whether they are one- or two-sided<br>*Only common tests should be described solely by name; describe more complex techniques in the Methods section.*
☒	☐	A description of all covariates tested
☐	☒	A description of any assumptions or corrections, such as tests of normality and adjustment for multiple comparisons
☐	☒	A full description of the statistical parameters including central tendency (e.g. means) or other basic estimates (e.g. regression coefficient) AND variation (e.g. standard deviation) or associated estimates of uncertainty (e.g. confidence intervals)
☒	☐	For null hypothesis testing, the test statistic (e.g. *F*, *t*, *r*) with confidence intervals, effect sizes, degrees of freedom and *P* value noted<br>*Give P values as exact values whenever suitable.*
☒	☐	For Bayesian analysis, information on the choice of priors and Markov chain Monte Carlo settings
☒	☐	For hierarchical and complex designs, identification of the appropriate level for tests and full reporting of outcomes
☐	☒	Estimates of effect sizes (e.g. Cohen's *d*, Pearson's *r*), indicating how they were calculated

Our web collection on statistics for biologists contains articles on many of the points above.

Software and code

Policy information about availability of computer code

Data collection	Data were acquired using MSTune Orbitrap Ascend version 4.1.4244 (Thermo Fisher Scientific)
Data analysis	UniDec v6.0.2 was used to analyze the DIA-PTCR data. BioPharmaFinder v5.0 Peptide Finder (Thermo Fisher Scientific) and PMi Biologic v4 (Protein Metrics, Inc) were used in the analysis of glycopeptide data.PMi Byomap v4.3 (Protein Metrics) was used for released glycan analysis. Chromeleon was used for data acquisition and analysis of 2-AA HILIC data.

For manuscripts utilizing custom algorithms or software that are central to the research but not yet described in published literature, software must be made available to editors and reviewers. We strongly encourage code deposition in a community repository (e.g. GitHub). See the Nature Portfolio guidelines for submitting code & software for further information.

Data

Policy information about availability of data

All manuscripts must include a data availability statement. This statement should provide the following information, where applicable:

- Accession codes, unique identifiers, or web links for publicly available datasets
- A description of any restrictions on data availability
- For clinical datasets or third party data, please ensure that the statement adheres to our policy

The data that support the findings of this study are available in the MassIVE repository under accession code MSV000092002. The FTP download link to be used during peer review is: ftp://MSV000092002@massive.ucsd.edu. The password is: UNIGLAMS. After publication, the direct link will be: ftp://massive.ucsd.edu/MSV000092002/. The PDB structure 3DLQ may be found at https://doi.org/10.2210/pdb3DLQ/pdb

Research involving human participants, their data, or biological material

Policy information about studies with human participants or human data. See also policy information about sex, gender (identity/presentation), and sexual orientation and race, ethnicity and racism.

Reporting on sex and gender	N/A
Reporting on race, ethnicity, or other socially relevant groupings	N/A
Population characteristics	N/A
Recruitment	N/A
Ethics oversight	N/A

Note that full information on the approval of the study protocol must also be provided in the manuscript.

Field-specific reporting

Please select the one below that is the best fit for your research. If you are not sure, read the appropriate sections before making your selection.

☒ Life sciences ☐ Behavioural & social sciences ☐ Ecological, evolutionary & environmental sciences

For a reference copy of the document with all sections, see nature.com/documents/nr-reporting-summary-flat.pdf

Life sciences study design

All studies must disclose on these points even when the disclosure is negative.

Sample size	We validated the reproducibility of the method using ovalbumin, a glycoprotein standard, and successfully reproduced the results in 5 replicates. The sample size of 5 was chosen for this standard as it provides sufficient evidence that the method reliably produces the same readout for the same sample and acquisition conditions. Sialidase treated Il22Fc were analyzed in triplicate by DIA-PTCR. IL22Fc activity was analyzed in duplicate
Data exclusions	No data was excluded from the study.
Replication	All attempts at replication were successful. We validated the reproducibility of the method using ovalbumin, a glycoprotein standard, and successfully reproduced the results in 5 replicates. Sialidase treated Il22Fc were analyzed in triplicate by DIA-PTCR. IL22Fc activity was analyzed in duplicate
Randomization	Randomization was not relevant to the study because samples were analyzed in a targeted manner, one at a time.
Blinding	Blinding was not relevant to the study as it was crucial to know what sample was being analyzed for proper mass assignment.

Reporting for specific materials, systems and methods

We require information from authors about some types of materials, experimental systems and methods used in many studies. Here, indicate whether each material, system or method listed is relevant to your study. If you are not sure if a list item applies to your research, read the appropriate section before selecting a response.

Materials & experimental systems

n/a	Involved in the study	
☐	☒	Antibodies
☐	☒	Eukaryotic cell lines
☒	☐	Palaeontology and archaeology
☒	☐	Animals and other organisms
☒	☐	Clinical data
☒	☐	Dual use research of concern
☒	☐	Plants

Methods

n/a	Involved in the study	
☒	☐	ChIP-seq
☒	☐	Flow cytometry
☒	☐	MRI-based neuroimaging

Antibodies

Antibodies used	goat anti-human IgG-horseradish peroxidase (HRP) antibody (Sigma, St Louis, MO c/n AP112P)
Validation	Detection substrate antibody: positive and negative controls used for binding assay, 3,3',5,5'-tetramethylbenzidine substrate solution (p/n T4444, Sigma-Aldrich, St Louis, MO).

Eukaryotic cell lines

Policy information about cell lines and Sex and Gender in Research

Cell line source(s)	In house CHO-K1 production cell line
Authentication	regulated manufacturing cell line for Genentech, not authenticated for this study
Mycoplasma contamination	Cell lines were not tested for mycoplasma contamination for this study
Commonly misidentified lines (See ICLAC register)	No commonly misidentified lines were used in this study

Plants

Seed stocks	No seed stocks were used in this study
Novel plant genotypes	no plant genotypes were used in this study
Authentication	no authentication was performed in this study
